# Natural variation in genome architecture among 205 *Drosophila melanogaster* Genetic Reference Panel lines

**DOI:** 10.1101/gr.171546.113

**Published:** 2014-07

**Authors:** Wen Huang, Andreas Massouras, Yutaka Inoue, Jason Peiffer, Miquel Ràmia, Aaron M. Tarone, Lavanya Turlapati, Thomas Zichner, Dianhui Zhu, Richard F. Lyman, Michael M. Magwire, Kerstin Blankenburg, Mary Anna Carbone, Kyle Chang, Lisa L. Ellis, Sonia Fernandez, Yi Han, Gareth Highnam, Carl E. Hjelmen, John R. Jack, Mehwish Javaid, Joy Jayaseelan, Divya Kalra, Sandy Lee, Lora Lewis, Mala Munidasa, Fiona Ongeri, Shohba Patel, Lora Perales, Agapito Perez, LingLing Pu, Stephanie M. Rollmann, Robert Ruth, Nehad Saada, Crystal Warner, Aneisa Williams, Yuan-Qing Wu, Akihiko Yamamoto, Yiqing Zhang, Yiming Zhu, Robert R.H. Anholt, Jan O. Korbel, David Mittelman, Donna M. Muzny, Richard A. Gibbs, Antonio Barbadilla, J. Spencer Johnston, Eric A. Stone, Stephen Richards, Bart Deplancke, Trudy F.C. Mackay

**Affiliations:** 1 Department of Biological Sciences, North Carolina State University, Raleigh, North Carolina 27595, USA;; 2 Laboratory of Systems Biology and Genetics, Institute of Bioengineering, School of Life Sciences, Ecole Polytechnique Fédérale de Lausanne (EPFL), CH-1015 Lausanne, Switzerland;; 3 Swiss Institute of Bioinformatics, 1015 Lausanne, Switzerland;; 4 Center for Education in Liberal Arts and Sciences, Osaka University, Osaka-fu, 560-0043 Japan;; 5 Genomics, Bioinformatics and Evolution Group, Institut de Biotecnologia i de Biomedicina (IBB), Department of Genetics and Microbiology, Campus Universitat Autònoma de Barcelona, 08193 Bellaterra, Spain;; 6 Department of Entomology, Texas A&M University, College Station, Texas 77843, USA;; 7 Genome Biology Unit, European Molecular Biology Laboratory (EMBL), 69117 Heidelberg, Germany;; 8 Human Genome Sequencing Center, Baylor College of Medicine, Houston, Texas 77030 USA;; 9 Virginia Tech Virginia Bioinformatics Institute and Department of Biological Sciences, Virginia Tech, Blacksburg, Virginia 24061, USA

## Abstract

The *Drosophila melanogaster* Genetic Reference Panel (DGRP) is a community resource of 205 sequenced inbred lines, derived to improve our understanding of the effects of naturally occurring genetic variation on molecular and organismal phenotypes. We used an integrated genotyping strategy to identify 4,853,802 single nucleotide polymorphisms (SNPs) and 1,296,080 non-SNP variants. Our molecular population genomic analyses show higher deletion than insertion mutation rates and stronger purifying selection on deletions. Weaker selection on insertions than deletions is consistent with our observed distribution of genome size determined by flow cytometry, which is skewed toward larger genomes. Insertion/deletion and single nucleotide polymorphisms are positively correlated with each other and with local recombination, suggesting that their nonrandom distributions are due to hitchhiking and background selection. Our cytogenetic analysis identified 16 polymorphic inversions in the DGRP. Common inverted and standard karyotypes are genetically divergent and account for most of the variation in relatedness among the DGRP lines. Intriguingly, variation in genome size and many quantitative traits are significantly associated with inversions. Approximately 50% of the DGRP lines are infected with *Wolbachia*, and four lines have germline insertions of *Wolbachia* sequences, but effects of *Wolbachia* infection on quantitative traits are rarely significant. The DGRP complements ongoing efforts to functionally annotate the *Drosophila* genome. Indeed, 15% of all *D. melanogaster* genes segregate for potentially damaged proteins in the DGRP, and genome-wide analyses of quantitative traits identify novel candidate genes. The DGRP lines, sequence data, genotypes, quality scores, phenotypes, and analysis and visualization tools are publicly available.

Studies in *Drosophila melanogaster* have revealed basic principles and mechanisms underlying fundamental genetic concepts of linkage and recombination and were instrumental in identifying canonical and evolutionarily conserved cell signaling pathways. Most *D. melanogaster* genes are evolutionarily conserved, leading to fly models for understanding common human diseases and behavioral disorders, dipteran disease vectors, and insects impacting agriculture, medicine, and forensics. Despite nearly a century of research on *D. melanogaster*, however, a large fraction of its coding and noncoding sequence has no known function (McQuilton et al. 2012). Recent efforts to induce mutations in every protein coding gene utilize transposable elements (Bellen et al. 2004, 2011), which have a different spectrum of allelic effects than SNPs and small insertions and deletions (indels). Comprehensive efforts to identify regulatory DNA elements in *Drosophila* (The modENCODE Consortium et al. 2010) have attributed functional effects to noncoding DNA, further complicating efforts to dissect the genotype-phenotype map. In addition, the vast majority of genetic analyses in *D. melanogaster* have used a few “wild type” strains representing a tiny sample of genetic diversity. Mutational effects in one genetic background are often enhanced or suppressed in other backgrounds (Mackay 2014). Such epistatic interactions provide a window for visualizing genetic interaction networks. In addition, *D. melanogaster* has a rich history as a model organism for population and quantitative genetics, generating inferences about regions under purifying natural selection independent of functional analyses and highlighting the contribution of common and rare variants in protein coding as well as regulatory sequences to the genetic architecture of complex traits (Flint and Mackay 2009; Mackay et al. 2009).

Efforts to utilize naturally occurring genetic variation in *D. melanogaster* to add to our understanding of functional DNA elements have been greatly expedited by the *Drosophila* Genetic Reference Panel (DGRP), a publicly available population of 205 sequenced inbred lines. Previously, we cataloged SNPs segregating in 168 DGRP lines (DGRP Freeze 1.0) (Mackay et al. 2012) and non-SNP variants in a subset of 39 lines (Massouras et al. 2012; Zichner et al. 2013). Here, we report the DGRP Freeze 2.0 with sequences of all lines and genotypes for SNP and non-SNP variants (indels, tandem duplications, and complex variants). We describe cytogenetic analysis of inversions, *Wolbachia* infection status, variation in genome size, molecular population genetics of indels and inversions, functional analyses of segregating variants, and online tools for association mapping of complex traits.

## Results

### Catalog of molecular polymorphism in the DGRP

We obtained Illumina sequences for 48 DGRP lines that were not sequenced previously or for which only 454 sequence data were available, as well as for six DGRP lines with low Freeze 1.0 coverage (Supplemental Data File S1). We aligned sequence reads to the *D. melanogaster* genome using BWA (Li and Durbin 2010) and Novoalign (Novocraft.com), recalibrated base quality scores, and locally realigned reads. The average mapped sequence coverage was 27× per line (Supplemental Data File S1).

**Figure 1.**
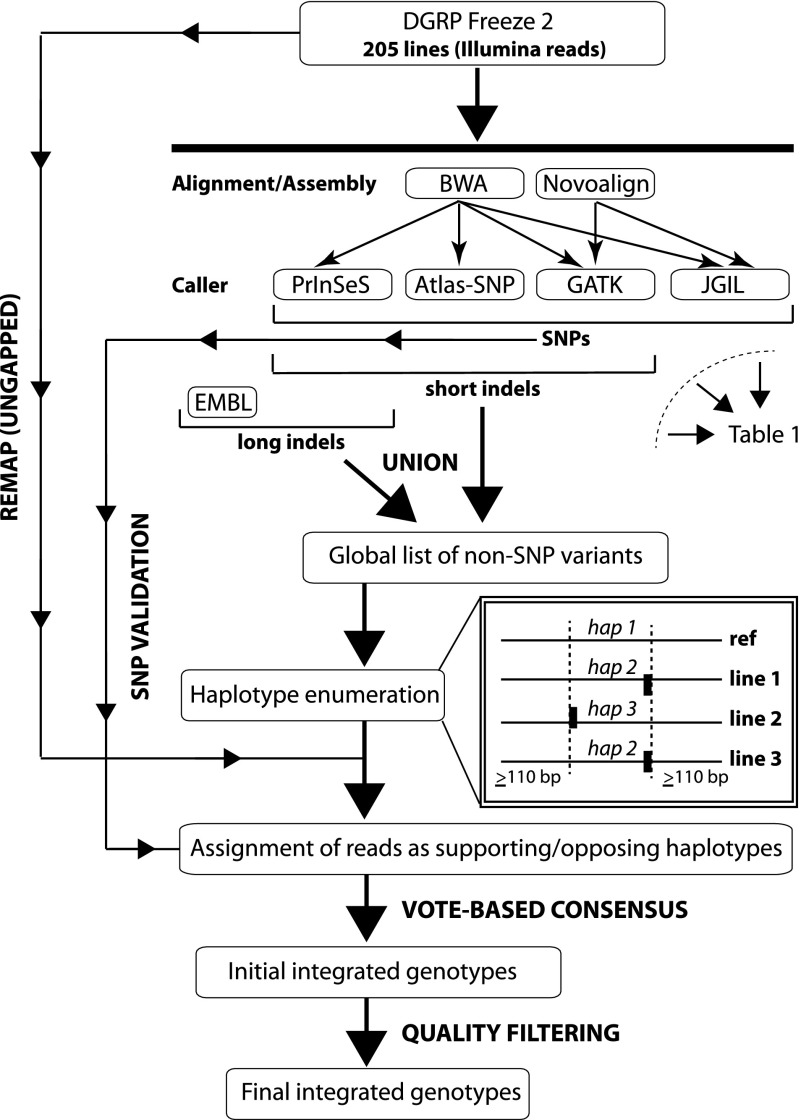
Flowchart of the integrated genotyping procedure used to call SNP and non-SNP variants. Seven different variant calling methods were used to derive a consensus list of variant calls. The variant calls were grouped into haplotype bins (indicated by dashed vertical lines) such that there is a region on both sides of each region containing two or more regions of at least 110 bp with no non-SNP variants in any line. The variable regions and their 110-bp flanking regions were used to derive the sequences of alternative haplotypes against which reads are aligned. Finally, reads were aligned and genotypes called, followed by quality filtering that accounted for the experimental design.

There are many algorithms for detecting SNP and non-SNP variants from short-read sequence data (Massouras et al. 2010; McKenna et al. 2010; Medvedev et al. 2010; Shen et al. 2010; Alkan et al. 2011; Rausch et al. 2012; Stone 2012). Identification of non-SNP variants is challenging with short reads (Onishi-Seebacher and Korbel 2011), since structural variants can produce alternative alignments and variant calls for the same variant. Methods combining several approaches to generate a consensus variant list give a lower false positive rate than individual methods (Mills et al. 2011; Zichner et al. 2013). Variant call quality can be further enhanced by genotyping to test if variants in the population are also present in the line under consideration (Waszak et al. 2010; Handsaker et al. 2011). In regions of low read depth, such genotyping may be possible even though variants cannot be discovered de novo. In this study, we used seven variant callers and integrated genotyping (Fig. 1) to comprehensively map genomic variation in 205 DGRP lines.

On average, the methods called over 580,000 SNPs, and 130,000 small (<100 bp) and 1400 large (≥100 bp) non-SNP variants per line; however, there was heterogeneity in the number of variants called by each method and the overall concordance among methods (Table 1). Therefore, we implemented an integrated genotyping algorithm, first using the combined data from all variant calling methods to update the genotypes of each DGRP line, then using the 205 variant call lists to genotype each DGRP line (Fig. 1). We identified 6,149,882 unique variants, including 4,853,802 SNPs and 1,296,080 non-SNP variants. The majority (98.28%) of the non-SNP variants were <100 bp.

**Table 1.**
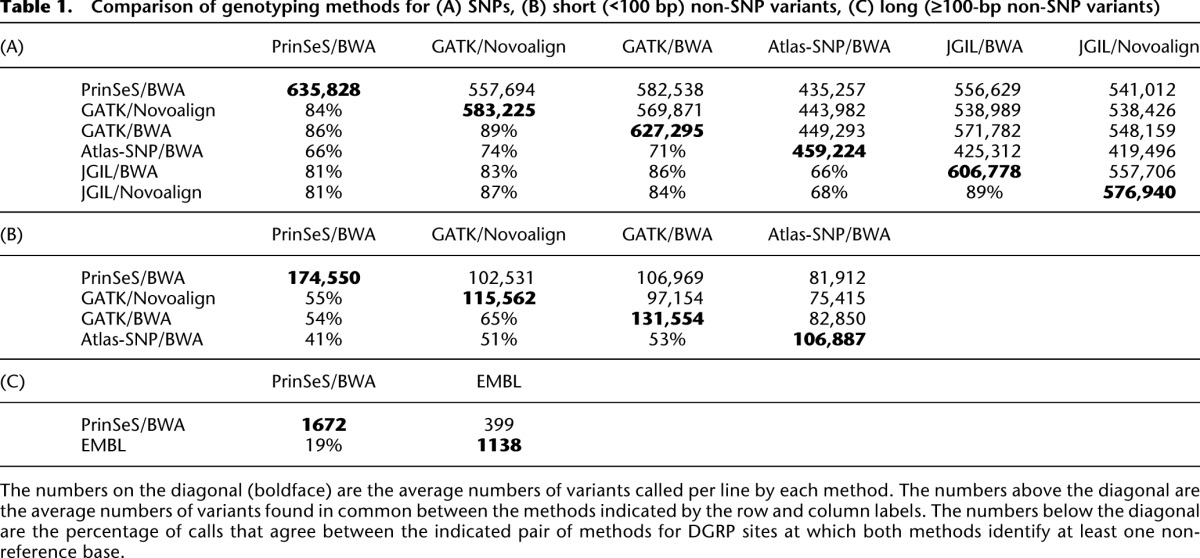
Comparison of genotyping methods for (A) SNPs, (B) short (<100 bp) non-SNP variants, (C) long (≥100-bp non-SNP variants)

### Validation of genotype calls

We used three strategies to validate genotype calls. First, we targeted 384 small (1–18 bp) indels affecting coding regions and 384 randomly chosen larger (30–313 bp) indels for validation by Sanger sequencing in five DGRP lines. A total of 315 small and 384 large indels were successfully assayed with Sanger technology for at least three lines. Of the 1463 small indel/line and 1876 large indel/line combinations with both Sanger and Illumina calls, 1458 (99.66%) and 1872 (99.79%), respectively, were concordant (Supplemental Data Files S2, S3).

Second, we performed high-density tiling microarray-based validation experiments using published data for six DGRP lines (Zichner et al. 2013) to assess the accuracy of the genotyping of larger deletions (>25 bp). We evaluated 3930 deletions ranging in size from 27 to 7533 bp. Of 5957 deletion/line comparisons, 5170 (86.8%) were true positives and 787 (13.2%) were false positives (Supplemental Fig. S1).

Third, we used the 454 sequence data from 38 lines (Mackay et al. 2012; Supplemental Table S1) to validate SNP and non-SNP calls. We used our integrated genotyping algorithm to call variants but restricted the input variant list to the final calls from the Illumina genotyping analysis. Using the same genotyping pipeline but a different sequencing chemistry serves to validate the Illumina data generation process. We used Fisher’s exact test to statistically evaluate whether the Illumina and 454 genotypes were concordant or discordant, using a nominal 5% significance threshold to declare discordance (Table 2; Supplemental Data File S4). Concordance was greater for homozygous than segregating Illumina calls for all variant types, was best for SNPs, and declined with increasing size of insertions and deletions. We conclude that our calls of homozygous SNP and small non-SNP genotypes, which comprise the vast majority of variants, are accurate and that large insertions and deletions should be independently confirmed using other methods.

**Table 2.**
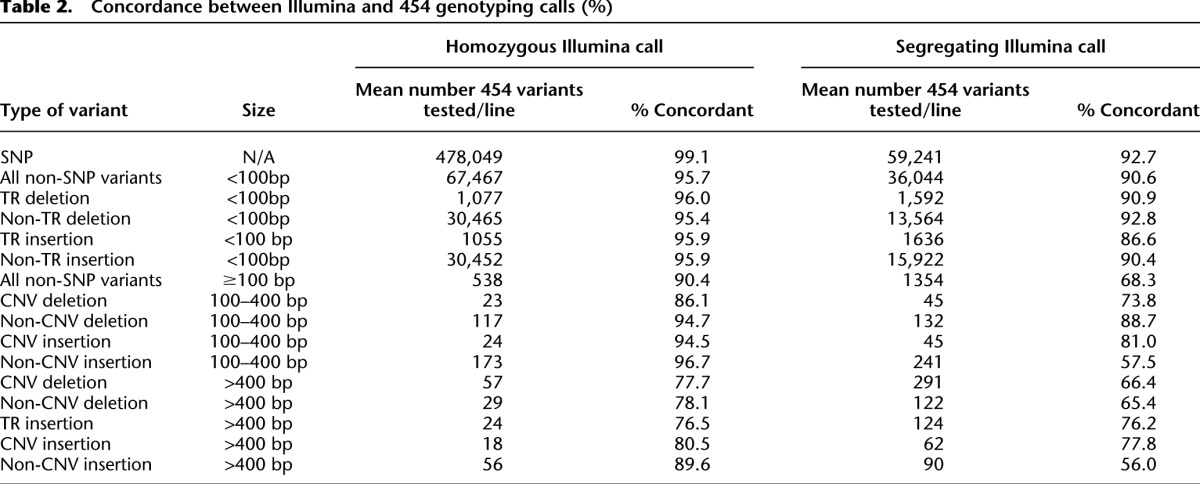
Concordance between Illumina and 454 genotyping calls (%)

We compared Freeze 2.0 variants and genotypes with the Freeze 1.0 SNP calls. Of the 5,222,888 polymorphic SNPs in the 158 lines with Freeze 1.0 Illumina data, 4,215,573 are present in the initial Freeze 2.0 call set. The reduction in number of SNP calls was mostly attributable to low frequency SNPs and/or SNPs near indels (Supplemental Fig. S2), suggesting that our integrated variant calling approach eliminated false SNPs near indels. Using a model tailored to the experimental design (Stone 2012), we generated quality scores for each of the 6,149,882 variants and for each genotype in each line. We filtered the genotypes based on the quality scores and limited all subsequent analyses to the 4,438,427 biallelic variants meeting the thresholds. For SNPs that were present in both freezes, the concordance rate between the homozygous genotypes was uniformly high (0.9988–0.9996) in all lines.

### Variation in numbers of segregating sites

The DGRP lines were derived by 20 generations of full-sib inbreeding and have an expected inbreeding coefficient of F = 0.986 (Falconer and Mackay 1996). Therefore, we expect that 1.4% of the variants will remain segregating, under the assumption of selective neutrality. Deleterious variants may be eliminated more rapidly than expected, while an increase in the number of segregating variants could occur from overdominant variants or from de novo mutations. Natural selection favoring heterozygotes can oppose fixation by inbreeding if there is true overdominance for fitness at individual loci or associative overdominance arising from complementary deleterious alleles that are closely linked in repulsion. If complementary deleterious alleles are embedded in polymorphic genetically divergent inversions, inversion heterozygotes may be polymorphic over the entire inverted region. Finally, the appearance of segregating sites can be generated if duplicate, divergent paralogous genes were mapped to a single gene of the pair.

**Figure 2.**
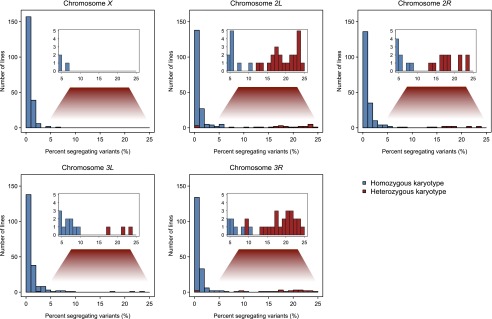
Distributions of the percent segregating variants in 205 DGRP lines, by chromosome. The distributions for homozygous standard or inverted karyotypes are given in blue, and the distributions for inversion/standard heterozygotes are given in red.

We assessed the number of segregating sites for each line by chromosome (Supplemental Data File S5) and found substantial variation in the number of segregating sites between and within chromosomes. Approximately 96% of the lines had 2% or fewer segregating X-linked variants, while on average 84% of the lines had 2% or fewer segregating autosomal variants (Fig. 2). Therefore, inbreeding was successful for the majority of variants. However, the distribution of the number of segregating sites on the autosomes was bimodal. In total, 62 of the 820 DGRP line/autosome combinations had ≥9% variants segregating; ≥20% variants remained segregating in 28 chromosomes (Supplemental Data File S5; Fig. 2).

### Inversion genotypes

*D. melanogaster* populations harbor polymorphic inversions (Stalker 1976; Mettler et al. 1977; Corbett-Detig and Hartl 2012). Recombination is suppressed between the inverted sequence and standard karyotype, leading to divergence between inversions and homo-sequential regions (Navarro et al. 1997, 2000; Andolfatto et al. 2001) and the potential for evolution of coadapted gene complexes (Kirkpatrick and Barton 2006; Hoffmann and Rieseberg 2008). A likely explanation for the large numbers of segregating autosomal variants in specific regions of some lines could be heterozygosity for inversions that are genetically divergent from the standard karyotype. Therefore, we determined inversion genotypes of the DGRP lines by cytogenetic analysis of polytene salivary gland chromosomes.

We identified 16 different segregating autosomal inversions (Table 3; Supplemental Data File S6). Of the 62 autosome/DGRP line combinations with >9% segregating sites, 60 had at least one heterozygous inversion, while two were the standard karyotype (Fig. 2). A possible explanation for the two exceptional karyotypes is that an inversion segregated in these lines when they were sequenced, but the standard karyotype was fixed in the interval between sequencing and the cytological analysis. Of the 758 autosome/DGRP line combinations with fewer than 9% segregating sites, 752 were homozygous for either the inverted or standard sequence (Fig. 2). However, six inversion heterozygotes (one for *In[3L]Y*, two for *In[3R]Mo*, and three for *In[2L]t*) had very low numbers of segregating sites. *In(3L)Y* is only present as a single heterozygote in the sample and could be of recent origin and hence not genetically differentiated from the standard karyotype. However, other chromosomes heterozygous for *In(3R)Mo* and *In(2L)t* had large numbers of segregating sites (Supplemental Data Files S4, S5). Possibly, these inversions do not have a single origin, and the old and new inverted sequences are segregating in the DGRP; or they could have undergone an even number of recombination events as heterokaryotypes, recovering a standard nucleotide configuration. Nevertheless, there is nearly a perfect correlation between large numbers of segregating sites and inversion heterozygosity (Fisher’s exact test *P* = 1.91 × 10^−81^).

**Table 3.**
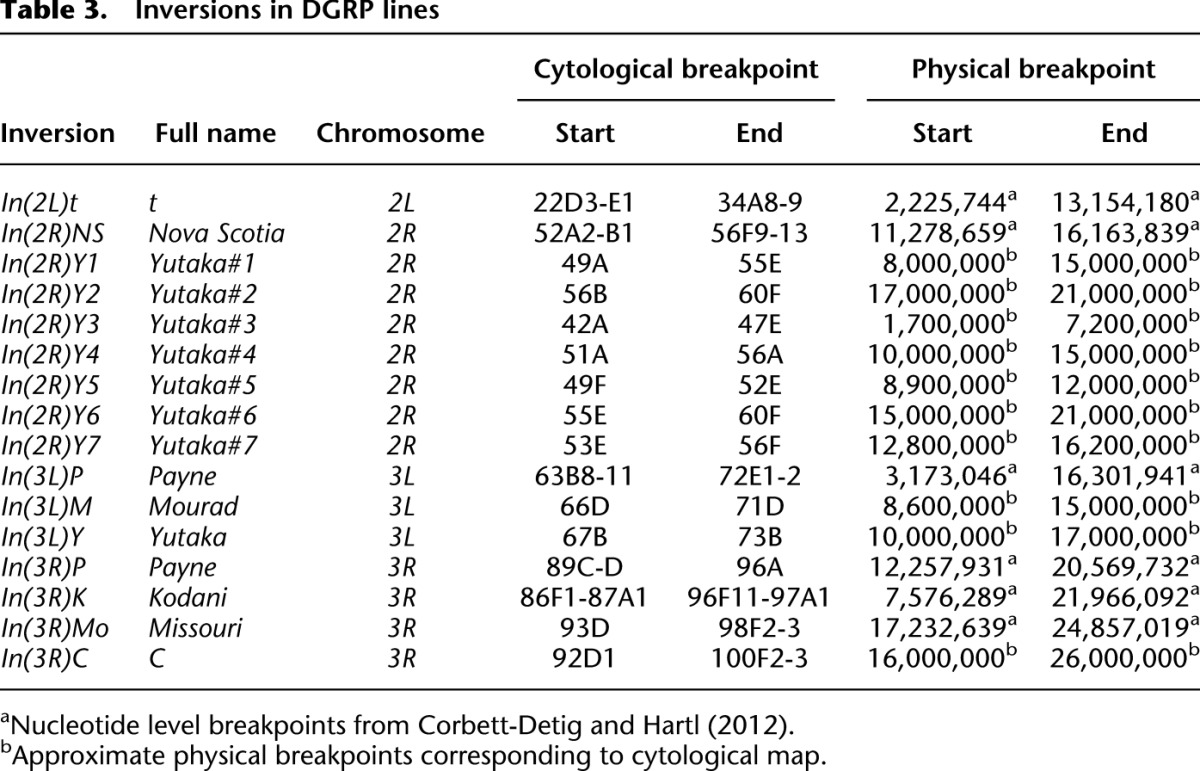
Inversions in DGRP lines

### *Wolbachia* infection

*Wolbachia pipientis* is a maternally transmitted endosymbiotic bacterium that infects ∼20% of all insects (Dunning Hotopp et al. 2007). *Wolbachia* can manipulate host biology to increase production of infected females, and hence its own transmission (Hoffmann et al. 1986). *D. melanogaster* populations worldwide are polymorphic for *Wolbachia* infection (Richardson et al. 2012). *Wolbachia* infection in *D. melanogaster* has been associated with resistance to infection by RNA viruses (Teixeira et al. 2008), but the full range of effects of *Wolbachia* on development, physiology, reproduction, and quantitative traits is unknown. We determined the *Wolbachia* infection status of the Freeze 2.0 DGRP lines, finding that ∼53% of the lines are infected (Supplemental Data File S7). *Wolbachia* sequences have been inserted into eukaryotic genomes (Dunning Hotopp et al. 2007). Therefore, we examined the DGRP lines for evidence of similar lateral gene transfer events and found that all infected lines had predicted insertions of ∼180-bp *Wolbachia* sequence at two genomic locations (Supplemental Fig. 3). However, PCR-based analyses revealed that only four DGRP lines contained the *Wolbachia* insertions (Supplemental Fig. S3). The insertions were incorrectly called in the remaining lines infected with *Wolbachia* because *Wolbachia* sequence reads were present for these lines, and the genotyping algorithm assigned them to the location to which they uniquely mapped in the four lines. This artifact did not occur for any other large insertions, all of which were either unique, as expected for a new *D. melanogaster* sequence present in DGRP lines but not the reference strain, or were homologous to other *D. melanogaster* sequences, as expected from insertions arising from transposable elements (TEs), local tandem duplications, and nonhomologous recombination.

### Variation in genome size

The large numbers of insertions and deletions suggest that the DGRP lines may vary in genome size. We estimated total genome size for each line using flow cytometry (Hare and Johnston 2011). There is significant variation in genome size (ANOVA *F*_204, 811_ = 2.61, *P* < 0.0001), ranging from 169.7 to 192.8 Mb (Supplemental Fig. S4; Supplemental Data File S8). Genome size differences were verified by the presence of double peaks in copreparations from lines with different average genome size (Ellis et al. 2014). The mean genome size of all lines (175.6 Mb) is close to that of the reference strain (175 Mb). The distribution is skewed toward the accumulation of large genomes, suggesting greater constraint on genome reduction than expansion.

Lines homozygous for *In(2R)NS*, *In(3L)P*, and *In(3R)K* and heterozygous for *In(3L)Y* had larger average genome sizes than the corresponding standard homozygous karyotypes, whereas lines homozygous or heterozygous for all other inversions had smaller average genome sizes than the standard karyotypes. We regressed genome size on the total number of “smaller” inversions and found a significant negative effect (*b* = −0.52, *F*_1,203_ = 8.25, *P* = 0.0045) (Supplemental Fig. S5). Although inversions account for only 4% of the variation in genome size, the magnitude of the effect is substantial at 0.5 Mb per inverted region.

### Population genomics of indels

Previously, we performed a population genomic analysis of SNPs in the DGRP Freeze 1.0 (Mackay et al. 2012). The SNP genotype calls are highly correlated between Freeze 1.0 and Freeze 2.0. Spearman rank order correlations (*ρ*) for estimates of SNP nucleotide polymorphisms (*π*) (Nei 1987) among 100-kb nonoverlapping windows range from *ρ* = 0.94 for the X chromosome to *ρ* = 0.99 for *3R* (Supplemental Table S1). Since population genomic inferences from analyses of SNP variation remain the same, we primarily focus here on indel variation.

We defined insertions and deletions in our variant calling algorithm with respect to the reference sequence. For population genetic inferences, we polarized insertion/deletion status evolutionarily with respect to *Drosophila simulans* and determined the ancestral and derived status of 210,268 biallelic indels. We found that 86% of “deletions” and 74% of “insertions” inferred from the reference genome were true deletions and insertions according to the polarized estimates.

Evolutionarily derived deletions (*n* = 145,015; 69%) outnumber insertions (*n* = 65,253; 31%) by 2.2:1 (Supplemental Table S2; Supplemental Fig. S6). This estimate is among the highest estimates of the deletion:insertion ratio for *D. melanogaster* but is consistent with previous estimates that indicate a bias toward higher deletion than insertion rates (Petrov 2002; Ometto et al. 2005; Assis and Kondrashov 2012; Leushkin et al. 2013). There are, on average, 60% fewer deletions (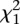 = 3815, *P* = 0) and 74% fewer insertions (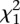 = 645.6, *P* = 0) on the X chromosome than on the major autosomal chromosomal arms (Supplemental Table S1), consistent with stronger selection against indels on the X chromosome. The observed bias toward deletions is not an artifact of the greater difficulty of calling large insertions than deletions. We called approximately equal numbers of insertions and deletions except for the largest variants, where we called more deletions than insertions relative to the reference (Table 2). Thus the calling bias is only for variants >400 bp. Since such variants are a very small fraction of the total, this bias cannot account for the excess of evolutionarily derived deletions.

Although most indels are small (1–2 bp), deletions are, on average, larger than insertions (Supplemental Table S2; Supplemental Fig. S6). However, the longest indels are insertions, most of which correspond to *P* transposable elements which have recently colonized the *D. melanogaster* genome (Kidwell 1993). Most large insertions are located in centromeric regions. The distributions of indel size are similar for 3′ and 5′ UTRs, large and small introns, and intergenic regions, while the size distribution of indels in coding regions has discrete “peaks” for indel sizes in multiples of 3 bp (Supplemental Fig. S7). This pattern suggests strong negative selection against frame-shifting indels compared to more relaxed selection for insertions and deletions spanning complete codons, a phenomenon previously reported for 39 DGRP lines (Massouras et al. 2012) and in humans (Montgomery et al. 2013).

The minor allele frequency (MAF) spectra (Supplemental Fig. S8) show an excess of low MAF indels compared to SNPs for all functional classes. Given that lower MAF variants are likely enriched for variants under purifying selection, these data are consistent with deleterious fitness effects of indels (Massouras et al. 2012). Insertions and deletions causing coding sequence frame-shifts are highly overrepresented among the low derived allele frequency (DAF) class (Supplemental Fig. S9), reinforcing the conclusion that negative selection is intense on this indel class. Relative to presumed neutral variants (synonymous SNPs and SNPs in small introns), all deletion classes have an excess of low-frequency derived alleles on all chromosomes. In contrast, the number of low-frequency derived insertion alleles is similar to or less than presumed neutral SNPs for insertions in small introns and nonframe shifting coding sequence insertions on the X chromosome. There is also a slight excess of high-frequency derived insertions compared to SNPs in all chromosomes and all functional categories except frame-shift insertions. This could indicate more positive selection on insertions than deletions.

These results suggest that natural selection acts differently on insertions and deletions, with stronger purifying selection on deletions (Petrov 2002; Assis and Kondrashov 2012; Leushkin et al. 2013). This is consistent with the mutational equilibrium theory for genome size evolution (Petrov 2002), where optimal genome size is maintained by purifying selection on small deletions and less selection on long insertions, compensating for sequence loss. This inference from population genomic analysis is consistent with the skewed distribution of genome sizes toward larger genomes.

### Nonrandom distribution of SNPs and indels

Previously, we found that SNP nucleotide polymorphism (*π*) in the DGRP was reduced near centromeres and telomeres and was positively associated with local recombination rate (for recombination rates < 2 cM/Mb) (Mackay et al. 2012). The pattern of *π*_*indel*_ along chromosomes is similar to that of SNP nucleotide diversity (Supplemental Fig. S10). There is a strong positive correlation between indel and nucleotide diversity for all chromosome arms (Supplemental Table S3; Massouras et al. 2012). Several biological mechanisms have been proposed for the clustering of SNPs and indels, which appears to be ubiquitous in prokaryotes and eukaryotes (Tian et al. 2008; Hodgkinson and Eyre-Walker 2011; McDonald et al. 2011; Jovelin and Cutter 2013). Possibly indels (Tian et al. 2008; Jovelin and Cutter 2013) and repeats (McDonald et al. 2011) are mutagenic because they induce error-prone DNA polymerase replication near the indel or repeat (Yang and Woodgate 2007); the regions in which SNPs and indels occur are inherently mutagenic; or SNPs and indels are subject to the same population genomic processes.

**Figure 3.**
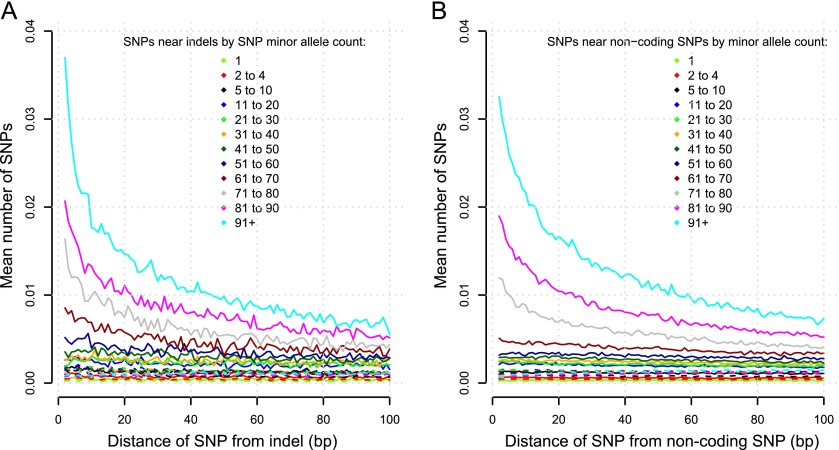
Nonrandom distribution of variants. The average number of SNPs (*y*-axis) for each distance in bp (*x*-axis) from either side of a variant of high frequency (MAF 40%–50%). Solid lines represent the number of SNPs of a given range of allele counts in lines that have the variant in question, whereas dashed lines show the number of SNPs in lines that do not have the variant. (*A*) Indels. (*B*) Noncoding SNPs.

To test the hypothesis that indels are mutagenic, we plotted the number of SNPs ± 100 bp from indels with MAF between 0.4 and 0.5, for different SNP minor allele counts. Intermediate-frequency SNPs are clustered near intermediate-frequency indels (Fig. 3A). Assuming intermediate-frequency indels are older than low-frequency indels, we expect enrichment for SNPs of all minor allele counts near them, since they would continuously generate new mutations. We did not observe this pattern (Fig. 3A). The same analysis for SNPs near intermediate-frequency noncoding focal SNPs also shows an elevated density of SNPs surrounding the focal SNPs (Fig. 3B), indicating that variant clustering is not unique to indel-containing regions. Thus, variant clustering is unlikely to be driven by indels. To test the hypothesis that regions containing increased polymorphism for SNPs and indels have elevated mutation rates, we performed similar analyses for the same regions, but using the lines that do not contain the focal indel alleles. The regions lacking indels contained fewer variants than those with the respective indels (Fig. 3), refuting the locally increased mutation rate hypothesis.

Evolutionary models of hitchhiking and background selection predict a positive correlation between recombination and polymorphism for all variants (Begun and Aquadro 1992; Charlesworth et al. 1993). We replicated our previous observation (Mackay et al. 2012) that SNP polymorphism is positively correlated with the local recombination rate, and extended this observation to insertions and deletions (Supplemental Table S3). Thus, local recombination rate affects the patterning of all types of variants, implicating evolutionary processes as the likely explanation for the observed clustering of variants. The lack of correlation between recombination and divergence for SNPs and indels (Spearman *ρ* = 0.037 genome-wide, *P* = 0.205) excludes mutations associated with recombination as the cause of the correlation between *π* and local recombination.

### Distribution of variants in chromatin domains

We determined enrichment or depletion of variants for five chromatin types (Supplemental Data File S9; Filion et al. 2010). Broadly expressed euchromatic genes that perform universal housekeeping functions are depleted of variants, consistent with purifying selection on these genes. Narrowly expressed euchromatic genes associated with more specific biological processes (Filion et al. 2010; van Steensel 2011) are enriched for variants, particularly in coding regions, suggesting that they are under less purifying selection and potentially more rapidly evolving than genes in other chromatin classes. Genes bound by Polycomb Group protein complexes and enriched for the repressive histone mark H3K27me3 are also enriched for variants, which is surprising because Polycomb-associated genes typically regulate developmental processes and are thought to be under strong purifying selection. Genes marked by Heterochromatin Protein 1 binding are located in pericentric regions and are strongly depleted for SNPs and small (<100 bp) indels, but enriched for larger (≥100 bp) indels, consistent with our observation that centromeric regions have reduced nucleotide and indel diversity and larger insertions. Interestingly, segmental duplications are highly biased toward centromeric regions in the human genome (She et al. 2004). The most prevalent type of repressive chromatin covers 48% of the genome and marks genes with low expression levels that are generally enriched for variants. While the chromatin classes were derived from one cell type and should be interpreted with caution, our results show that variants are nonrandomly distributed with respect to the chromatin state of the underlying DNA sequence.

### Population genomics of inversions

Levels and patterning of polymorphism are affected by the recombinational landscape and natural selection, both of which are different for regions bearing chromosomal inversions (Navarro et al. 1997; Andolfatto et al. 2001). Recombination is reduced in inversions and is pronounced near the breakpoints of paracentric inversions such that the sequence immediately adjacent to the inversion breakpoint rarely recombines. Recombination is also reduced in inversion heterozygotes because single recombination events within the inverted region lead to inviable aneuploid gametes. However, genetic exchange still occurs in inverted segments from multiple recombination events and/or gene conversion. Thus, we expect young inversions to have reduced genetic diversity but little divergence from their standard karyotype progenitor, while regions harboring older inversions will separately accumulate mutations in the standard and inverted sequences that lead to differentiation between them. We expect polymorphism to be less within inversion karyotypes, and genetic differentiation to be greater between inversion karyotypes in the regions proximal to the breakpoints than the more central regions of the inverted sequence (Navarro et al. 2000).

**Figure 4.**
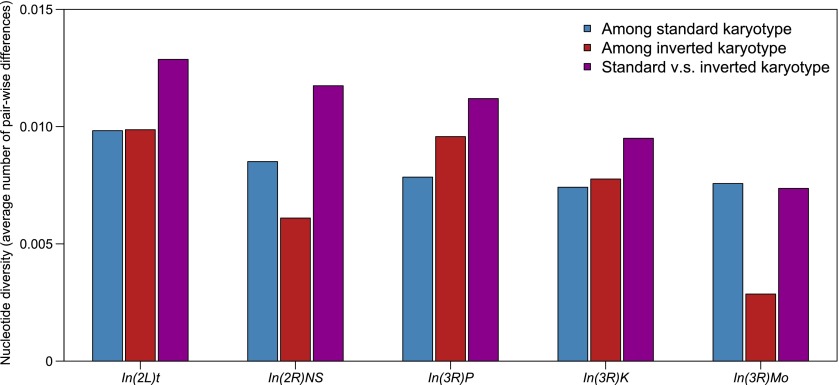
Nucleotide diversity (π) within standard karyotypes (blue bars), within inverted karyotypes (red bars), and between standard and inverted karyotypes (purple bars) within genomic regions encompassed by common polymorphic inversions. The calculation was based on nonmissing genotypes only, with indels (>1 bp) or multiple nucleotide polymorphisms receiving the same weight as SNPs regardless of their length.

Our observation that lines polymorphic for inverted and standard karyotypes have large numbers of segregating sites indeed implies that the inverted and standard karyotypes are genetically divergent. We calculated *π* for the inverted regions within lines with inverted and standard karyotypes, as well as between the inverted and standard karyotypes (Fig. 4). In all cases, the divergence between karyotypes is higher than the average nucleotide diversity within standard and inverted karyotypes (Fig. 4). However, local variation in polymorphism and diversity swamps any signal of reduction in polymorphism within and increase in diversity between inversion karyotypes near the breakpoints relative to the central regions (Supplemental Fig. S11).

### Functional annotation of segregating variants

We annotated functional consequences (Supplemental Table S4) of individual segregating variants, identifying 6637 potentially damaging variants that affect splice donor or acceptor sites, cause frame-shift mutations, loss of start or stop codons, or lead to premature stop codons. Collectively, they affect 3868 genes in at least one DGRP line. The allele frequency distribution of these potentially damaging variants is shifted to the lower end of the frequency spectrum relative to those of less damaging variants (Supplemental Fig. S12), as expected if they have deleterious fitness effects.

Next, we identified closely linked cosegregating variants that might ameliorate these potentially damaging variants (Gan et al. 2010). We found pairs of compensatory variants (SNPs that rescue a premature stop codon variant and indels in the same genes that compensate each other to avoid frame-shifts) in an average of 50 genes per line and a total of 403 compensated genes in all lines. These compensatory variants are largely in close physical proximity (1–2 bp) and in near complete linkage disequilibrium (D′ ∼ 1) (Supplemental Fig. S13). In all cases, variants that would otherwise introduce a premature stop codon are present only in lines carrying the compensatory variants. Given their close proximity, recombination events are unlikely to occur between pairs of adjacent compensated variants. This suggests that the compensatory variants at these codons most likely occurred first in the population, thus allowing the second mutation to occur without introducing a stop codon. Consistent with our inferred timeline of mutations, these compensated variants segregate at higher frequency in the DGRP than other potentially damaging variants (Supplemental Fig. S14).

**Figure 5.**
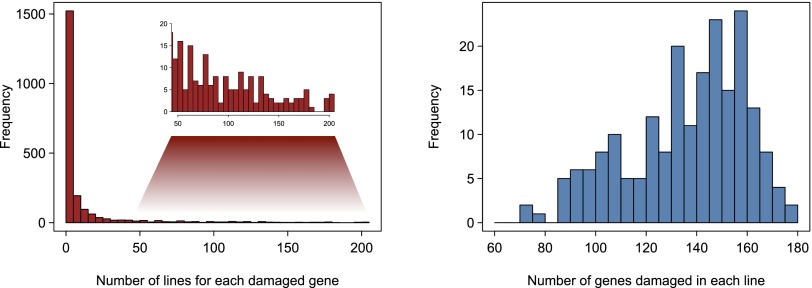
Histograms of the numbers of DGRP lines containing each damaged gene (*left*) and the number of damaged genes per DGRP line (*right*).

Finally, we performed gene-centric annotation by integrating all sequence variations overlapping coding regions in each DGRP line to take into account the widespread occurrence of multiple variants in single genes. We found 2169 genes whose proteins are damaged by the combination of all variants in them in at least one DGRP line (∼15% of *Drosophila* protein coding genes) (Supplemental Data File S10). On average, each of these affected genes is damaged in ∼13 of the 205 DGRP lines, and each line contains ∼136 potentially damaged genes (Fig. 5). These potentially damaging variants and genes are a new source of novel mutations for functional analyses. Gene ontology enrichment analysis showed that multigene families affecting chemosensation, detoxification of xenobiotic substances, immune and defense response, and proteolysis are enriched for damaged genes (Supplemental Data File S11). The same gene families are rapidly evolving along the *Drosophila* phylogeny (*Drosophila* 12 Genomes Consortium 2007).

### Genetic relationships among DGRP lines

**Figure 6.**
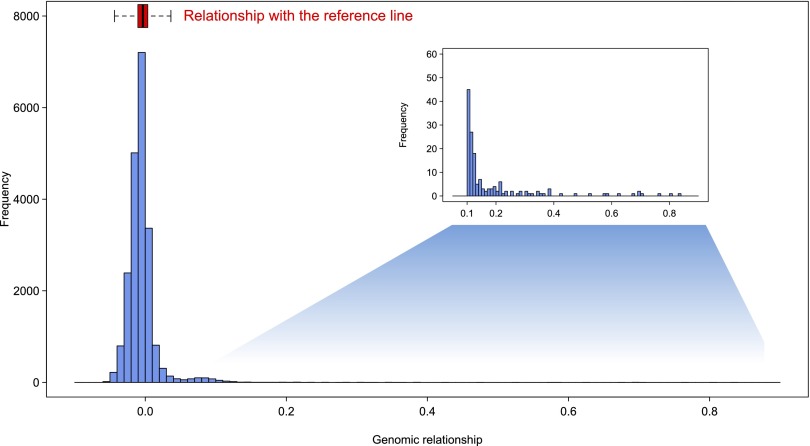
Histogram of genomic relationships among DGRP lines (20,910 possible pairs). The distribution of the relationship between all DGRP lines and the reference sequence is displayed as a box plot.

Genetic diversity is highly elevated between inverted and standard karyotypes in the region of the inversion. Thus, we expect that individuals of the same inversion karyotype will be more related to each other than to individuals of the standard karyotype. Therefore, we quantified patterns of genetic relatedness among the DGRP lines by constructing the genetic relationship matrix between all pairs of DGRP lines (Supplemental Fig. S15; Van Raden 2008; Ober et al. 2012). The distribution of relatedness is bimodal with the major peak centering around zero and the vast majority of pairwise relatedness within the range of distance to the reference strain (Fig. 6). The minor peak consists of 567 pairs (2.7% of all possible pairs) with relatedness greater than 0.05. There are 11 pairs (0.05% of all possible pairs) among 16 lines that have a genomic relationship greater than 0.5. Therefore, most DGRP lines are unrelated, consistent with sampling from a large, randomly mating population. However, some lines have higher genomic relatedness due to cryptic genetic relatedness (Astle and Balding 2009), possibly caused by sampling siblings from the natural population and/or shared inversion karyotypes.

**Figure 7.**
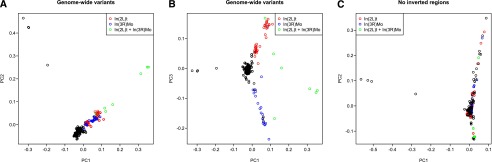
Principal component analysis of DNA sequence variation in the DGRP. Principal components (PCs) are computed using EIGENSTRAT. (*A*) PC plot of PC1 versus PC2. (*B*) PC plot of PC1 versus PC3. (*C*) PC plot of PC1 versus PC2 after PCs were recomputed excluding all variants in regions encompassing major inversions (*In[2L]t*, *In[2R]NS*, *In[3R]P*, *In[3R]K*, *In[3R]Mo*). With the exception of four highly related pairs of lines, there is no apparent clustering of karyotype groups.

Principal component (PC) analysis reveals clusters of related lines that carry major inversions. The first two PCs separate lines carrying both *In(2L)t* and *In(3R)Mo* from all other lines (Fig. 7A), while the first and third PCs discriminate lines with *In(2L)t* from those with *In(3R)Mo* (Fig. 7B). The PC clustering by inversions disappears when variants within the inverted regions are excluded (Fig. 7C). Lines with the same inversions are more related to each other than are lines homozygous for the standard karyotype (Supplemental Fig. S16), confirming that the PC clusters are driven by increased average genomic relationships within inversions.

We also computed genomic relationships separately for each chromosome arm (Supplemental Fig. S17). The chromosome-wide relationships among the lines are specific to each arm and are different from the genome-wide pattern (Supplemental Fig. S17). The genomic heterogeneity of relatedness among chromosomal arms suggests that population structure other than the known inversions is likely minimal; otherwise, inter-chromosomal correlation of relatedness would arise.

### Linkage disequilibrium

**Figure 8.**
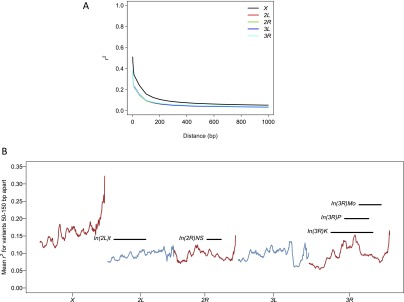
Patterns of LD. (*A*) Decay in LD with physical distance, by chromosome arm. (*B*) Genome-wide spatial variation in LD. Mean *r*^*2*^ between variants within 50–150 bp of each other in sliding windows (in 100-kb steps) of 1 Mb is plotted.

We assessed pairwise linkage disequilibrium (LD) between polymorphic variants using the *r*^2^ parameterization (Hill and Robertson 1966). Average LD decays rapidly as the distance between the variants increases, and the rate of decay is substantially lower on the X chromosome than autosomes (Fig. 8A), consistent with previous observations based on fewer DGRP lines and SNP variants only (Mackay et al. 2012). There is substantial variation in local LD along the genome (Fig. 8B). In general, LD near centromeres and telomeres is significantly greater than in other chromosomal regions.

**Figure 9.**
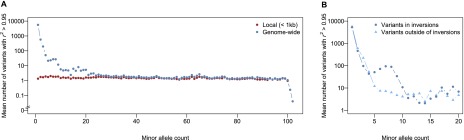
Relationship between LD and minor allele count. For each of the minor allele counts, 1000 random variants are sampled, and the mean number of variants genome-wide or locally (<1 kb) in strong LD (*r*^*2*^ > 0.95) with the focal variant is calculated. (*A*) Relationship between the mean number of variants in strong LD with the focal variant and minor allele count. (*B*) Relationship between the mean number of variants in strong LD with the focal variant and minor allele count, stratified according to the location of the focal variant (within or outside of inversions).

The rapid decline in local LD with physical distance is favorable for identifying causal genes and possibly variants in genome-wide association (GWA) studies using the DGRP. However, long-range LD could significantly impair our ability to identify QTLs. For each of 1000 randomly sampled variants with a specified number of minor alleles in the population, we counted variants that are in strong LD (*r*^2^ > 0.95) with it locally (within 1 kb) and genome-wide. There are consistently very few (mean = 1.43) variants in high local LD with the focal variant. However, the number of long-range variants in high LD with focal variants depends on the minor allele count of the focal variant and can be in the thousands for very low frequency variants (Fig. 9). Although local LD does not seem to differ for the regions with or without inversions (Fig. 8B), long-range LD as measured by the number of nonlocal variants in high LD is greater for variants within inversions (Fig. 9). Therefore, GWA studies based on individual variants should be restricted to common polymorphisms and also take into account inversions.

### Associations between quantitative traits, *Wolbachia*, inversions, and genome size

The range and magnitude of effects of *Wolbachia* infection and segregating inversions on organismal phenotypes is not known. Therefore, we assessed to what extent inversion genotypes and *Wolbachia* infection status are associated with starvation resistance, startle response, time to recover from chill coma (Mackay et al. 2012), resistance to acute (Weber et al. 2012) and chronic (Jordan et al. 2012) oxidative stress, several sleep phenotypes (Harbison et al. 2013), and olfactory behavior (Swarup et al. 2013). The effect of *Wolbachia* is only significant for acute and chronic resistance to oxidative stress (Supplemental Table S5). *In(3R)K* is associated with starvation resistance in females and acute oxidative stress resistance in males; *In(2L)t*, *In(2R)NS*, and *In(3R)Mo* are associated, often strongly and in a sex-specific manner, with sleep traits; and *In(2L)t* and *In(3R)Mo* are associated with olfactory behavior in both sexes (Supplemental Table S5). The DGRP lines vary significantly in genome size, which could also affect variation in quantitative trait phenotypes. However, correlations of quantitative traits with genome size were small for all traits and not significant in any analysis.

### Genome-wide association analyses in the DGRP

Prior to performing GWA analyses using the DGRP, we must adjust the phenotypic data to account for cryptic genetic relatedness, effects of inversions (lines with the same inversion karyotype have higher relatedness, and there is elevated LD within inverted regions) and *Wolbachia* infection status. Association tests can be performed for individual variants or by gene. The former can identify putative causal alleles but is restricted to the 1,920,276 variants with minor allele frequencies ≥0.05 to avoid spurious associations due to LD caused by limited sample size (Fig. 9). Gene-based tests can interrogate the remaining variants with low allele frequencies, which should contribute substantial variation if variation in the trait is maintained by mutation-selection balance (Turelli 1984), and can also evaluate effects of common variants and all variants. However, they are sensitive to the exact methods used for weighting variants within a gene (Madsen and Browning 2009; Han and Pan 2010; Wu et al. 2010, 2011; Lee et al. 2012). In either scenario, we perform associations on the adjusted phenotypic values using a model that accounts for cryptic relatedness among the lines. For single marker association, we use a mixed model that incorporates the relationship matrix, whereas for the gene-based tests, we add covariates corresponding to the major principal components that account for relatedness. We performed GWA analyses for starvation resistance, a classic quantitative trait, *Wolbachia* infection status (in this case, the data were not corrected for *Wolbachia* infection), and genome size (Supplemental Data Files S12, S13; Supplemental Text S1).

The need to adjust for *Wolbachia* and inversions and account for relatedness is illustrated by quantile-quantile plots (Supplemental Fig. S18) from single variant GWA analysis of starvation resistance in females, which is associated with *In(3R)K* (Supplemental Table S5). Unadjusted data show substantial systematic inflation of test statistics, while adjusting for *Wolbachia* and inversions and accounting for relatedness using a mixed model significantly alleviate the inflation. The top associations for the individual and gene-based tests for all three traits are only partially overlapping, highlighting the complementary nature of these tests. Only a few variants/genes reached conservative Bonferroni-adjusted significant thresholds, and all suggest novel candidate genes affecting the traits. Examples include a SNP in *genghis khan* (*gek*, a protein kinase), associated with female starvation resistance, and SNPs in *pointed* (*pnt*, a transcription factor) and *CG32521* (a gene of unknown function), associated with genome size. *myotubularin* (*mtm*), which is involved in chromosome segregation and the mitotic cell cycle (McQuilton et al. 2012), is a plausible candidate gene associated with genome size and reached Bonferroni-level significance in the gene-based tests of association with this trait.

## Discussion

Here, we present a molecular polymorphism map for 205 sequenced inbred *D. melanogaster* lines comprising Freeze 2.0 of the DGRP. We utilized seven different algorithms for detecting variants to produce a consensus variant list, and further fine-tuned the variant calls for each line using an integrated genotyping strategy that borrows power from the variant calls in all lines. We further provide quality scores for all 4,853,802 SNP and 1,296,080 non-SNP variants using a method that takes into account the experimental design used to generate the DGRP. Independent validation of variant calls gives low false positive rates for SNPs and small (<100 bp) indels, which comprise >98% of all variants. We performed a cytogenetic analysis of large segregating inversions, genotyped all lines for the presence of the maternally transmitted *Wolbachia* endosymbiont, and estimated genome size by flow cytometry. These data provide a comprehensive characterization of natural variation in genome architecture in this powerful genetic model organism that can be used to gain insights about natural selection and the evolution of genome size, and enhance the functional annotation of the *D. melanogaster* genome. We also describe improved statistical methodology for genome-wide association mapping of quantitative traits in a scenario where all variants are known and the rapid decay in LD with physical distance enables high-resolution mapping.

Our molecular population genomic analysis of evolutionarily polarized deletion and insertion variants showed that deletions outnumber insertions by a ratio of greater than 2:1, consistent with previous studies on smaller data sets, suggesting a bias toward the deletion mutation rate in *Drosophila* (Petrov 2002; Assis and Kondrashov 2012; Leushkin et al. 2013). Site frequency spectra show an excess of low-frequency polymorphisms compared to SNPs for insertions and deletions from all functional categories but especially for frame-shifting indels, implicating strong purifying selection against these variants. However, the site frequency spectra suggest stronger selection on deletions than insertions, which could lead to the maintenance of an optimal genome size (Petrov 2002). Our direct observation of variation in genome size in the DGRP, which varies by ∼14%, is in accord with this hypothesis. This variation in genome size is similar to that observed for an *Arabidopsis thaliana* population in Sweden (Long et al. 2013). The distribution of genome size variation is skewed toward larger genomes, consistent with stronger purifying selection against deletions than insertions.

As observed previously (Tian et al. 2008; McDonald et al. 2011; Massouras et al. 2012; Jovelin and Cutter 2013), we found a strong positive correlation between the genomic distribution of indels and SNPs. These correlated patterns of polymorphism are, in turn, correlated with local recombination, suggesting that the nonrandom distributions are due to hitchhiking and background selection (Begun and Aquadro 1992; Charlesworth et al. 1993). Alternative explanations that indels are mutagenic or that the highly polymorphic regions have high mutation rates were not supported by our analyses.

Inversions are islands of genomic divergence in this *D. melanogaster* population. Nucleotide diversity is elevated between inverted and homo-sequential genomic regions relative to the average diversity of inverted and standard regions, and consequently, lines heterozygous for inversions have large numbers of segregating sites in the region encompassed by the inversion. There is a greater extent of long-range LD within inverted sequences than the same regions on the standard karyotypes, indicative of lower recombination rates and effective population sizes of inversions. It is intriguing that variation in genome size is significantly associated with inversions. The mechanistic basis of increased or decreased genome size in the different inversion karyotypes is an open question for future study. Previously, we inferred that there was little global population structure in the DGRP from our eigen-decomposition of the genetic covariance matrix, but noted that the large variance in this decline did not preclude local structure due to structural variation (Mackay et al. 2012). Here, we performed a more comprehensive analysis of variation in genetic relatedness in the DGRP and showed that individuals with the same inversion karyotype are more related to each other than to individuals of the standard karyotype, accounting for most of the variation in relatedness among the DGRP lines and local structure. Inversions can harbor “coadapted gene complexes” associated with fitness (Dobzhansky 1937), and indeed, many fitness-related traits have been associated with inversion polymorphism in *Drosophila* species (Hoffmann and Rieseberg 2008). We showed that variation in starvation and oxidative stress resistance, sleep traits, and olfactory behavior are all associated with inversion polymorphism, and future evaluation of more quantitative traits in the DGRP will provide a detailed picture of effects of inversions on complex traits.

Lateral gene transfer of *Wolbachia* sequences into insect genomes is common, most likely because its presence in developing gametes is a favorable scenario for germline integration (Dunning Hotopp et al. 2007). Lateral gene transfer is a potential mechanism for the acquisition of novel genes, but to date has not been reported for *Wolbachia* sequences in *D. melanogaster*. We identified two different insertions of small *Wolbachia* insertions in four DGRP lines. Future analyses of the transcriptomes of these lines will reveal whether the insertions are transcribed and potentially functional. The forces maintaining *Wolbachia* infection in *D. melanogaster* populations near 50% remain mysterious. Although infection status has been associated with resistance to infection by RNA viruses (Teixeira et al. 2008), effects of *Wolbachia* infection on the quantitative traits assessed in the DGRP are rarely significant.

The goal of the Berkeley *Drosophila* Genome Project (BDGP) Gene Disruption Project (Bellen et al. 2004, 2011) is to generate mutations in all *D. melanogaster* genes as tools for functional analysis, and that of the *Drosophila* modENCODE Project (The modENCODE Consortium et al. 2010) is to identify sequence-based functional elements in *Drosophila*. The DGRP complements these efforts. The millions of molecular variants segregating in the DGRP are novel mutations for functional analysis and represent a different functional class from the transposon-tagged mutations produced from the BDGP Gene Disruption Project. Indeed, 15% of all *D. melanogaster* genes segregate for potentially damaged proteins in the DGRP, yet these damaged genes are compatible with live, fertile flies (at least under standard laboratory conditions). Molecular population genomic analyses using the DGRP highlight genomic regions under purifying selection, complementing modENCODE functional motifs. GWA analyses of quantitative traits in the DGRP provide new functional annotation of the *D. melanogaster* genome by identifying novel candidate genes associated with these traits. These genes typically have well-described effects on other traits, play key roles in early developmental processes, or are computationally defined genes with no known function, but have never been associated with the focal trait. Subsequently, the full power of *Drosophila* genetics can be applied to validating marker-trait associations: mutations, RNAi constructs, and outbred QTL mapping populations (Huang et al. 2012; Jordan et al. 2012; Weber et al. 2012; Harbison et al. 2013; Swarup et al. 2013). The future of understanding the genetic architecture of quantitative traits lies in our ability to progress from one-gene-at-a-time associations to understanding how entire genetic and transcriptional networks causally affect complex organismal phenotypes. The DGRP is an ideal resource for systems genetics (Ayroles et al. 2009; Massouras et al. 2012) and epistatic interaction network analyses (Yamamoto et al. 2009; Huang et al. 2012; Swarup et al. 2013) of molecular and complex organismal traits.

The DGRP lines, sequence data, genotypes, quality scores, and phenotypes are publicly available. The DGRP website (http://dgrp2.gnets.ncsu.edu) hosts an updated pipeline for single marker GWA analysis which accounts for effects of *Wolbachia* infection and major inversions as well as cryptic relatedness among the DGRP lines; a new genome browser track for visualizing individual line genotypes and functional annotations for any specified genomic region; and all published phenotypes. These data will be useful for testing new analytical methods as well as for teaching general principles of population and quantitative genetics.

## Methods

### DGRP lines

We established isofemale lines from gravid females collected in Raleigh, NC, and inbred them by 20 generations of full-sib mating, followed by random mating (Mackay et al. 2012). All flies were reared and all phenotypes assessed under standard culture conditions (cornmeal-molasses-agar-medium, 25°C, 60%–75% relative humidity, 12-h light-dark cycle) unless otherwise specified.

### DNA isolation, library construction, and sequencing

We extracted genomic DNA from ∼500–1000 flies per DGRP line using the Gentra Puregene Tissue Kit (Qiagen) and purified the samples by phenol-chloroform extraction. We constructed high molecular weight double-strand genomic DNA samples into Illumina paired-end libraries according to the manufacturer’s protocol (Illumina) (Supplemental Text S2) and sequenced shotgun DNA libraries on the Illumina HiSeq 2000 or GAII platforms, according to the manufacturer’s specifications.

### Sequence read mapping and initial genotyping

We aligned Illumina sequence reads to the Dmel 5.13 reference genome (http://flybase.org) with BWA (v0.5.9-r16) (Li and Durbin 2010) and Novoalign (Novocraft.com) using default parameters. We used GATK (v1.0.5506) (McKenna et al. 2010) software to remove duplicate sequence reads, recalibrate base quality scores, and locally realign regions around indels for BWA alignments (DePristo et al. 2011). We excluded positions with >2000 coverage and mapped reads with *phred* scores <25 and/or mapping quality <10. We applied GATK (v1.0.5506) (McKenna et al. 2010) and JGIL (Stone 2012) to the BWA and Novoalign alignments, and Atlas-SNP (Shen et al. 2010) and PrinSeS (Massouras et al. 2010) to the BWA alignments to genotype SNPs. We genotyped non-SNP variants <100 bp using GATK, Atlas-SNP, and PrinSeS. We genotyped non-SNP vaiants ≥100 bp using PrinSeS, DELLY (Rausch et al. 2012), Pindel (v0.2.4d) (Ye et al. 2009), CNVnator (v0.2.2) (Abyzov et al. 2011), and Genome STRiP (v1.0.4) (Handsaker et al. 2011) as described in Zichner et al. (2013).

### Integrated genotyping

We performed integrative genotyping in two stages. First, we genotyped each line separately using all SNP and non-SNP variants from the output of the individual variant calling methods to provide the alternative haplotypes from which to choose variants. In the second stage, we again performed genotyping for each line, using the 205 variant lists resulting from the first stage (Supplemental Text S2). The resulting 6,149,882 nonredundant variants were then assigned variant and genotype quality scores using JGIL (Stone 2012). We retained for subsequent analyses nonoverlapping biallelic variants whose *phred* scale quality scores were at least 500 and genotypes whose sequencing depths were at least one and genotype quality scores at least 20. The final VCF genotype file (http://dgrp2.gnets.ncsu.edu) containing 4,438,427 variants gives the number of supporting and opposing reads for each variant in each line, genotypes with the maximum posterior probability, and the corresponding quality scores (Stone 2012).

### Validation

We used three strategies to validate genotype calls. First, we performed Sanger sequencing for 384 small (1–18 bp) indels affecting coding regions and 384 larger (30–313 bp) randomly chosen indels on five DGRP lines (DGRP_304, DGRP_324, DGRP_354, DGRP_355, DGRP_395). Second, we used previously published data (Zichner et al. 2013) on genomic DNA hybridization to Affymetrix GeneChip *Drosophila* 1.0R tiling arrays for six DGRP lines (DGRP_208, DGRP_304, DGRP_313, DGRP_315, DGRP_437, DGRP_555) and the reference strain to validate deletions >25 bp (Supplemental Text S2). Finally, we used 454 sequence (Roche) data from 38 DGRP lines (Mackay et al. 2012) to validate SNP and non-SNP calls (Supplemental Text S2). We used our integrated genotyping algorithm to count supporting and opposing reads of alleles for variants and tested the allele counts from Illumina and 454 for concordance using a Fisher’s exact test.

### Inversion karyotypes

We assessed inversion genotypes by cytogenetic analysis of polytene salivary gland chromosomes of third instar larvae by staining with lactic-acetic orcein. We identified inversions by comparison to the standard map of Bridges (1935). We initially examined two larvae from each DGRP line and subsequently confirmed inversion heterozygotes or segregating inversions by examining additional larvae and/or F1 hybrids of the DGRP line with the standard Canton S karyotype.

### *Wolbachia* status

We used PCR to determine the infection status of each line with respect to the endosymbiont, *Wolbachia pipientis* (Braig et al. 1998; Richardson et al. 2012; Supplemental Text S2). We used DGRP_101 and DGRP_105 as negative controls and DGRP_142 and DGRP_149 as positive controls. We also developed PCR assays to genotype all DGRP lines for insertions of *Wolbachia* genome at *2R*:16,594,660 and *2R*:19,117,791 (Supplemental Text S2). We purified PCR products for lines positive for *Wolbachia* insertions using the Zymo Clean and Concentrator kit (Zymo Research Corporation) and subjected them to Sanger sequencing using the ABI 3730XL platform.

### Genome size

We estimated genome sizes for 1016 individual females (at least three individuals per line) using flow cytometry with *Drosophila virilis* (1C = 328 Mb) as an internal standard, as described in Hare and Johnston (2011) but with a final concentration of propidium iodide stain at 25 mg/mL. The estimate of genome size was the proportion of stain uptake (expressed as a channel number by the flow cytometer) of the sample relative to the standard times the amount of DNA in the standard. We calculated the average genome size and standard deviation of genome size and performed additional replicate measurements as needed to produce a standard error of 0.5%. We tested whether the differences in genome sizes were true by flow cytometry analysis of copreparations of females from lines with different average genome size. We evaluated the association of segregating inversions with variation in genome size using the ANOVA model *Y* = *μ* + *G* + *ε*, where *Y* is the standard deviation of genome size within a line and *G* is the number of segregating inversions (0, 1, 2, 3, or 4) within lines.

### Population genomics

We used 357,708 JGIL-filtered, biallelic indels present in at least 101 lines to conduct the indel population genomics analyses. We assigned indels to one of six functional classes (coding sequence, 5′ and 3′ UTR, long [>100 bp] and short [≤100 bp] introns, intergenic sequence) using the 5.49 version annotations of the *D. melanogaster* reference genome (Marygold et al. 2013). We discarded indels spanning more than one functional class, leaving 357,608 indels with a valid functional class. We analyzed insertions and deletions separately, after first polarizing ancestral and derived states with respect to the high quality second-generation assembly genome of *D. simulans* (Hu et al. 2013) as an outgroup (Supplemental Text S2). We inferred the derived allele status for 210,268 indels. We manually checked a sample of 500 derived indels to which our polarizing protocol was applied; all were correct. Therefore, we conclude that the specificity of our procedure is very high, although we excluded 41% of the original indel data set from our evolutionary analyses.

We used *π*_*indel*_ to describe indel polymorphism, a measure analogous to nucleotide diversity (*π*), which does not take into account indel size. We used an analogous measure to estimate divergence (*k*) (Librado and Rozas 2009). We estimated fixed indel divergence for *D. melanogaster, D. simulans*, and *D. yakuba* using the multiple alignments *D. melanogaster* Oct. 2006 from the VISTA Browser (Frazer et al. 2004). We estimated these diversity measures for the whole genome and by chromosome arm (*X*, *2L*, *2R*, *3L*, *3R, 4*) in 100-kb nonoverlapping windows. We also estimated the minor allele frequency (MAF) distribution for indels and the derived allele frequency (DAF) distributions for both deletions and insertions. We used the nonparametric Spearman rank correlation coefficient (*ρ*) to test for covariation among the diversity estimates. We used the recent high resolution recombination map of *D. melanogaster* (Comeron et al. 2012) to correlate recombination with the diversity measures.

### Functional annotation of variants

We annotated the functional consequences of variants on annotated genes (FlyBase R5.49) (Marygold et al. 2013) using SnpEff (v3.1m) (Cingolani et al. 2012). We considered variants annotated as SPLICE_SITE_ACCEPTOR, SPLICE_SITE_DONOR, START_LOST, FRAME_SHIFT, STOP_GAINED, STOP_LOST to be “potentially damaging” for the affected proteins. We also performed a line-specific annotation integrating all homozygous variants each line carries. For each gene, we translated the variant transcript using the standard genetic code and compared the variant protein to the reference protein using the global alignment “stretcher” utility in EMBOSS (v6.5.7) (Rice et al. 2000). We considered the variant protein to be potentially damaged if the START or STOP codon was lost or the sequence identity with the reference protein was smaller than 90%. We considered a gene to be potentially damaged if all of its splice variants were affected.

### Analysis of relatedness and population structure

We calculated the realized genome-wide relationship matrix **G** among all DGRP lines using biallelic common variants (MAF > 0.05) with a call rate >80%. This computation was performed using the Van Raden (2008) formula implemented in the rrBLUP R package (v4.0) (Endelman 2011). The relationship matrix was normalized by the mean value of the diagonal elements. For analysis of population structure, we performed a principal component analysis (PCA) using EIGENSTRAT (v4.2) (Price et al. 2006). We pruned LD using the LD pruning utility in PLINK (v1.07) (Purcell et al. 2007) such that in a moving window of every 500 variants, the maximum pairwise *r*^*2*^ was smaller than 0.2. We excluded variants within 2 Mb of the major inversions (*2L*:0.4Mb-14.9Mb, *2R*:9Mb-18Mb, *3R*:6Mb-27Mb) from this analysis. We tested the significance of the top eigenvalues using the Tracy-Widom statistic implemented in EIGENSTRAT.

### Variant-based association mapping

We performed genome-wide association studies in two stages. In the first stage, we adjusted the data for the effects of *Wolbachia* infection and major inversions [*In(2L)t*, *In(2R)NS*, *In(3R)P*, *In(3R)K*, and *In(3R)Mo*] based on mean phenotypic values of each line. We then used the adjusted line means to fit a linear mixed model in the form of *y* *=* **X***b* *+* **Z***u* *+ e*, where *y* is the adjusted phenotypic values, **X** is the design matrix for the fixed SNP effect *b*, **Z** is the incidence matrix for the random polygenic effect *u*, and *e* is the residual. The vector of polygenic effects *u* has a covariance matrix in the form of **A***σ*^2^, where *σ*^2^ is the polygenic variance component. We fitted this linear mixed model using the FastLMM program (v1.09) (Lippert et al. 2011).

### Gene-based association mapping

We performed a burden test and a nonburden sequence kernel association test (SKAT) to assess the cumulative effect of all variants within one kilobase of each annotated gene. The weighted burden test weights the contribution of each variant in a gene by the reciprocal of the standard deviation of its estimated minor allele frequency and uses the weighted averages to estimate a score statistic (Madsen and Browning 2009; Han and Pan 2010). The SKAT kernel function builds a relationship matrix detailing relatedness of individuals based upon all variants within a gene. This relationship matrix is fit as the covariance matrix of a random effect in a linear mixed model framework and used to estimate a variance component score to discern the significance of a trait association (Wu et al. 2011). The SKAT kernel function used was linear and did not up-weight the relative contribution of minor alleles.

We performed both the weighted burden test and SKAT using the SKAT package (Wu et al. 2011) in R v3.0.1 (R Development Core Team 2013). For both methods, male and female starvation resistance and genome size were fit with an identity link function and fixed effect covariates for *Wolbachia* infection status, major inversions, and the 11 principal components explaining the most genetic variation in the DGRP (Tracy-Winom *P*-value < 0.01). *Wolbachia* infection status was fit with a logit link function in a likewise manner, excluding the fixed effect of *Wolbachia* infection status. We performed gene-based tests for all variants, and for common (MAF ≥ 0.05) and rare (MAF < 0.05) variants separately.

## Data access

The DGRP lines are available from the Bloomington *Drosophila* Stock Center (http://flystocks.bio.indiana.edu/Browse/DGRP.php) (see Supplemental Data File S1 for stock numbers). Raw sequence data have been submitted to the NCBI Sequence Read Archive (SRA; http://www.ncbi.nlm.nih.gov/sra) under accession numbers listed in Supplemental Data File S1, and to the Baylor College of Medicine Human Genome Sequencing Center (https://www.hgsc.bcm.edu/arthropods/drosophila-genetic-reference-panel). The genotypes, quality scores, phenotypes, and web-based analysis tools are available from the DGRP website (http://dgrp2.gnets.ncsu.edu).
